# *Lactobacillus* Regulates *Caenorhabditis elegans* Cell Signaling to Combat *Salmonella* Infection

**DOI:** 10.3389/fimmu.2021.653205

**Published:** 2021-03-08

**Authors:** Mengzhou Zhou, Xiaozhen Liu, Hai Yu, Joshua Gong

**Affiliations:** ^1^National “111” Center for Cellular Regulation and Molecular Pharmaceutics, Key Laboratory of Fermentation Engineering (Ministry of Education), School of Food and Biological Engineering, Hubei University of Technology, Hubei, China; ^2^Guelph Research and Development Centre, Agriculture and Agri-Food Canada, Guelph, ON, Canada; ^3^Engineering Research Center of Health Food Design & Nutrition Regulation, School of Chemical Engineering and Energy Technology, Dongguan University of Technology, Dongguan, China

**Keywords:** *Lactobacillus*, *Salmonella*, *Caenorhabditis elegans*, cell signaling, antimicrobial peptides, defense molecules

## Abstract

*Salmonella typhimurium* DT104 infection causes the death of *Caenorhabditis elegans*, which can be prevented by certain *Lactobacillus* isolates. However, the molecular mechanisms of both the host response to the infection and the protection by *Lactobacillus* are largely unclear. The present study has investigated the life-span and gene expression of both wild-type (WT) and mutants in some key components of cell signaling in response to *S. typhimurium* infection and protection from *Lactobacillus zeae*. The results indicated that the gene expression of *daf-16* in the DAF/ insulin-like growth factor (DAF/IGF) pathway, *ced-3* and *ced-9* in the programmed cell death (PCD) pathway, *lys-7, spp-1*, and *abf-3* for antimicrobial peptide production, and *bar-1* involved in the production of other defense molecules was all significantly upregulated when the wild-type (WT) was subjected to DT104 infection. On the contrary, the gene expression of *tir-1, sek-1*, and *pmk-1* in the p38 mitogen-activated protein kinase (MAPK) pathway and *clec-60, sod-3*, and *skn-1* for the production of other defense molecules was significantly suppressed by DT104. Pretreatment of the worms with *L. zeae* LB1 significantly upregulated the expression of almost all the tested genes except for *ced-3, ced-9, abf-2, age-1*, and *dbl-1* compared with the nematode infected with DT104 only. Mutants defective in the cell signaling or other defense molecules of *C. elegans* were either more susceptible (defective in *nsy-1, sek-1, pmk-1, ced-3, ced-9, skn-1*, or *daf-16*) or more resistant (defective in *age-1* or *dbl-1*) to DT104 infection than the WT except for the mutant defective in *sod-3*. Mutants defective in antimicrobial peptides (*lys-7* or *abf-3*) were also more susceptible than the WT. In contrast, the mutant defective in *spp-1* became more resistant. When all the mutants were pretreated with *L. zeae* LB1, five mutants that are defective in *nsy-1, sek-1, pmk-1, abf-3*, or *lys-7* showed no response to the protection from LB1. These results suggest that *L. zeae* LB1 can regulate *C. elegans* cell signaling including the p38 MAPK pathway and downstream production of antimicrobial peptides and defense molecules to combat *Salmonella* infection.

## Introduction

Probiotics have long been used to improve animal/human health. Antagonizing enteric infections and enhancing host immune responses are among the crucial benefits of probiotics (1, 2). Probiotics are normally commensal bacteria in the mammalian gastrointestinal tract. Probiotics and their products have important roles in gut homeostasis and the functions of innate and adaptive immune systems (3, 4). *Salmonella* is a well-known enteric pathogen that causes human food- and water-borne illnesses and even death. Development of effective control strategies and technologies, including the use of probiotics, has been extensively studied in the past (5–7). A highlight from these studies is the application of *Caenorhabditis elegans* as an animal model for efficient selection of probiotic candidates (8–10).

*Caenorhabditis elegans* is a small soil nematode that can consume bacteria as its food. It has been used extensively as an experimental model to study bacterium and host interactions due to its short reproductive life cycle, clear genetic background, ease of culturing and genetic manipulation, and availability of mutants (11–13). Although the nematode lacks many defense mechanisms presented in higher organisms, it still has complexity and specificity responding to different bacteria at the level of immune regulation, innate immunity in particular, which makes it suitable for elucidating the molecular mechanisms between bacterium and host interactions (14). Previous studies have shown that the worms can be infected and killed by different bacterial pathogens, including *Pseudomonas aeruginosa* (11), *Salmonella enterica* (15, 16), *Staphylococcus aureus* (17), *Enterococcus faecalis* (12), and enterotoxigenic *Escherichia coli* (ETEC; 18). In addition, a broad overlap of the bacterial virulence factors required for pathogenesis has been found between mammals and *C. elegans* (12, 18). Thus, *C. elegans* has increasingly been used to help screen the efficacy of probiotics for pathogen control (8, 9, 19, 20). By using *C. elegans*, we have previously identified several *Lactobacillus* isolates with the ability to antagonize *Salmonella* infection to the nematode (9). The protection offered by one of the isolates, named *Lactobacillus zeae* LB1, has been found to be mediated by a neurotransmitter dopamine through regulation of cell signaling in *C. elegans* (21). Furthermore, this isolate has demonstrated the ability to attenuate *Salmonella* infection in the spleen and liver of broiler chickens and reduce *Salmonella* SPI-1 virulence gene expression in the chicken cecum (22). However, the molecular mechanisms underlying the protective effects, including the regulation of cell signaling, remain to be further elucidated.

It is known that *C. elegans* immune defense mechanisms are evolutionarily conserved, including the DAF/insulin-like growth factor (DAF/IGF) pathway, p38 mitogen-activated protein kinase (p38 MAPK) pathway, the transforming growth factor-β (TGF-β) signaling pathway and the programmed cell death (PCD) pathway (23–27). The *C. elegans* innate immune response consists of the production of numerous antimicrobial proteins, many of which are produced from genes that are induced upon pathogen infection (28–30). Moreover, the expression of different putative antimicrobials involved in the defense of both nematodes and mammals against infection by different pathogens can be regulated by signaling pathways (28, 29, 31). By investigating the life-span of *C. elegans* and the corresponding gene expression of key components in its cell signaling and defense pathways, including the production of antimicrobial peptides and other defense molecules when exposed to *Salmonella typhimurium* DT104 and *Lactobacillus*, this study has determined that *L. zeae* LB1 regulates *C. elegans* cell signaling pathways to combat *Salmonella* infection. The results are reported herein.

## Materials and Methods

### *Caenorhabditis elegans* and Bacteria

*Caenorhabditis elegans* N2 Bristol wild-type and mutants that are defective in *lys-7* (mutant ok1384), *nsy-1* (mutant ag3), *pmk-1* (mutant km25), *sek-1* (mutant ag1), *skn-1* (mutant zu67), *dbl-1* (mutant nk3), *spp-1* (mutant ok2703), *abf-3* (mutant ok3366), *daf-16* (mutant mu86), *age-1* (mutant hx546), *sod-3* (mutant gk235), *ced-9* (mutant n1950), or *ced-3* (mutant n717), and the double mutant *ced-9;ced-3* (mutant n2812/n717) were obtained from Caenorhabditis Genetics Center (CGC), University of Minnesota, Minnepolis, USA. *Caenorhabditis elegans* strains were routinely maintained on nematode growth medium (NGM) plates seeded with *E. coli* OP50 using standard procedures (32).

*S. typhimurium* DT104 is a porcine multiantibiotic-resistant isolate (33). This strain was cultured on tryptic soy broth (TSB) or tryptic soy agar at 37°C for 16 h. Following three washes with M9 medium, 200 μl of cell suspension (10^8^ CFU/ml) was spread on a NGM plate (100 mm in diameter) and dried for 3 h at 22°C before beginning of the life-span assay. *L. zeae* LB1 was grown in de Man Rogosa Sharpe (MRS) broth or on MRS agar at 37°C for 18–24 h in an anaerobic chamber (Coy Laboratory Products, Grass Lake, MI) with an atmosphere of 85% N_2_, 10% CO_2_, and 5% H_2_ (9). After three washes with M9 medium, 200 μl cell suspension of *L. zeae* LB1 (10^8^ CFU/ml) was spread on a NGM plate (100 mm in diameter) and dried for 3 h at 22°C prior to the use.

### Life-Span Assay of *C. elegans*

The life-span assays of *C. elegans* were performed using the published methods with some modifications (8, 19, 34). Briefly, the synchronized *C. elegans* were transferred to NGM agar with *E. coli* OP50 and incubated at 25°C for 48–60 h until they reached the L4 stage. In the assays to evaluate the protective effect of *L. zeae* LB1 on nematodes, 50 of L4 stage worms were transferred onto the agar plates seeded with either *E. coli* OP50 or LB1 followed by incubation at 25°C, which was designated as day 0. After 18 h incubation worms on each plate were transferred to a fresh NGM plate daily that were seeded with DT104 and incubated at 25°C. In parallel, worms within the control group were transferred to a fresh NGM plate daily that had been seeded with *E. coli* OP50 after the 18 h incubation with the same bacterium. The survival of nematode was examined at 24-h intervals up to 15 days. To determine the survival of *C. elegans*, the number of live worms was recorded daily, and the percentage of surviving worms was calculated by the following formula: survival (%) = (live worms/total worms used) × 100. A worm was considered to be dead when it failed to respond to touch. In assays where a mutant was examined, the procedure remained unchanged. Each assay was repeated at least twice unless it is otherwise indicated.

### RNA Extraction

Approximately 150 worms were sampled from each treatment on day 2 of the life-span assays and were then subjected to RNA extraction. The nematodes were washed and disrupted using the method described previously (34) before RNA extraction with the mirVana miRNA Isolation Kit. The RNA integrity was determined by visualization in an agarose gel after treating with DNase I (Ambion, TX) followed by verified as DNA-free by PCR assays. The RNA concentration was determined with a NanoDrop ND-1000 spectrophotometer (NanoDrop Technologies, Wilmington, DE, USA).

### Reverse Transcription and Quantitative PCR Analysis

*Caenorhabditis elegans* gene expression was determined by quantitative PCR (QPCR) analysis after reverse transcription using SuperScript first-strand synthesis system (Invitrogen, Carlsbad, CA, USA). Two housekeeping genes Gapdh and Act-1 were used as internal controls. QPCR assays were performed using 7,500 Real Time PCR System (Applied Biosystems, Foster, CA, USA) and brilliant SYBR green QPCR master mix (Bio-Rad Laboratories, Richmond, VA) following the program: 5 min at 95°C and 40 cycles of 95°C for 30 s, 56°C for 1 min, and 72°C for 30 s. For QPCR assays, each tube contained 12.5 μl Master Mix, 3.75 μl each of the primers at 150 nM, 1 μl cDNA sample, and 4 μl irradiated and double autoclaved dH2O. The PCR primers are listed in Table 1.

**Table 1:** Primers of QPCR assay^*^.

**Primer**	**Amplicon (bp)**	**Sequence (5^**′**^to 3^**′**^)**	**Source or Reference**
Act-1-F	121	CCCCACTCAATCCAAAGGCT	(34)
Act-1-R		GTACGTCCGGAAGCGTAGAG	
Daf-16-F	181	TCGTCTCGTGTTTCTCCAGC	(34)
Daf-16-R		TAATCGGCTTCGACTCCTGC	
Age-1-F	359	CTCCTGAACCGACTGCCAAT	(34)
Age-1-R		AAATGCGAGTTCGGAGAGCA	
Lys-7-F	153	GTACAGCGGTGGAGTCACTG	(34)
Lys-7-R		GCCTTGAGCACATTTCCAGC	
Clec-60-F	219	CGGTTTCAATGCGGTATGGC	(34)
Clec-60-R		TGAAGCTGTGGTTGAGGCAT	
Clec-85-F	121	CCAATGGGATGACGGAACCA	(34)
Clec-85-R		CTTCTGTCCAGCCAACGTCT	
Abf-3-F	189	AACAGATTGGGGTCAGCTCG	(34)
Abf-3-R		TGGAGACCATTATTGCCGGG	
Spp-1-F	106	TGGACTATGCTGTTGCCGTT	(34)
Spp-1-R		ACGCCTTGTCTGGAGAATCC	
Abf-2-F	176	CCGTTCCCTTTTCCTTGCAC	(34)
Abf-2-R		GACGACCGCTTCGTTTCTTG	
Tir-1-F	223	TTGGGTGCACAAAGAGCTGA	(34)
Tir-1-R		GGTCGGTGTCGTTCTGTTCA	
Nsy-1-F	122	AGCGGCTCGATCAACAAGAA	(34)
Nsy-1-R		CCCATTCCACCGATATGCGA	
Sek-1-F	158	CACTGTTTGGCGACGATGAG	(34)
Sek-1-R		ATTCCGTCCACGTTGCTGAT	
Pmk-1-F	115	CCAAAAATGACTCGCCGTGA	(34)
Pmk-1-R		CTTTTGCAGTTGGACGACGA	
Bar-1-F	119	CATGGTAGTCCGCGACTTGT	(34)
Bar-1-R		CGAGAATTGACCAGCTCCAGA	
Skn-1-F	153	CTGGCATCCTCTACCACCAC	(34)
Skn-1-R		TTGGTGATGATGGCCGTGTT	
Dbl-1-F	194	TTTTGCGGCGAACAAATCGT	(34)
Dbl-1-R		TTCGCTGTTGCCTGTTTGTG	
Ced-3-F	167	AGAAGGAGCTTGCTAGAGAGGA	This study
Ced-3-R		ACTGCTTTCACGATCTCCCG	
Sod-3-F	88	GAAGATCGCCACCTGTGCAA	This study
Sod-3-R		CAAGTAGTAGGCGTGCTCCC	
Ced-9-F	146	GTCTAATCTCGTTCGGCGGT	This study
Ced-9-R		CCAGCTCCGATTGTGTTCCT	
Gapdh-F	158	ACTCGACCCACGGTCAATTC	(21)
Gapdh-R		ACTCGACAACGAAATCGGCT	

* *All the PCR products amplified with the pairs of primers designed in this study have been verified by DNA sequencing*.

The target gene expression was calculated using the 2^−ΔΔCt^ method (35). The ΔCt represents the difference between the Ct value with the primers to a target gene and the Ct value to the housekeeping genes. The ΔΔCt represents the difference between the ΔCt value of treatment group (either treated with Lactobacillus or *Salmonella*) and the ΔCt value of control group (treated with *E. coli* OP50). The values derived from 2^−ΔΔCt^ represent fold changes of samples in abundance relative to the reference samples. The reference samples (treated with *E. coli* OP50) had the 2^−ΔΔCt^ value of 1.

## Results

### Enhancement in the Resistance of *C. elegans* to DT104 Infection by LB1

**Figure 1:**
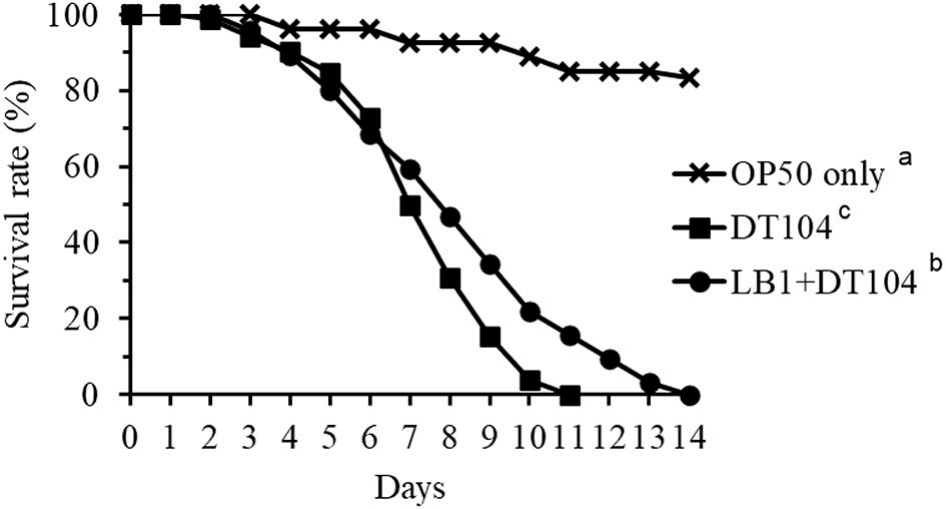
Effect of pretreatment with isolate *Lactobacillus zeae* LB1 on the survival of *Caenorhabditis elegans* infected with *Salmonella typhimurium* DT104. The worms were first fed either *Escherichia coli* OP50 or LB1 at 10^8^ CFU/mL for 18 h and then DT104 for the remaining days. Treatments: ■, *E. coli* OP50 and then DT104; •, LB1 and then DT104; ×, *E. coli* OP50 only. All the groups showing different letters were significant different (*P* ≤ 0.05) in their survival curves.

Figure 1 shows the effect of isolate LB1 on the life-span of *C. elegans* infected with *S*. *typhimurium* DT104. Although pretreatment of the WT nematode (N2) with isolate *L. zeae* LB1 did not eliminate death caused by DT104, LB1 pretreatment significantly extended (*P* ≤ 0.05) the life-span of the worms infected with DT104 only. These results were similar to our previously reported observations with a temperature-sensitive mutant (SS104) of *C. elegans* for preselection of probiotic candidates (9).

### Response of the WT *C. elegans* in Gene Expression to DT104 Infection With or Without LB1 Pretreatment

To examine the host immune response against *S*. *typhimurium* DT104 infection, the WT worms on day 2 of the life-span assay were selected based on the observation that the numbers of viable worms started to decrease on days 3–5 (Figure 1). The major components in the p38 MAPK (*tir-1, nsy-1, sek-1*, and *pmk-1*), DAF/IGF (*daf-16* and *age-1*), PCD (*ced-3* and *ced-9*) pathways, previously identified antimicrobial peptides *(lys-7, spp-1, abf-2, clec-85, clec-60*, and *abf-3*), and other reported defense molecules (*sod-3, dbl-1, skn-1*, and *bar-1*) were used as indicators for host response at the level of signaling transduction.

**Figure 2:**
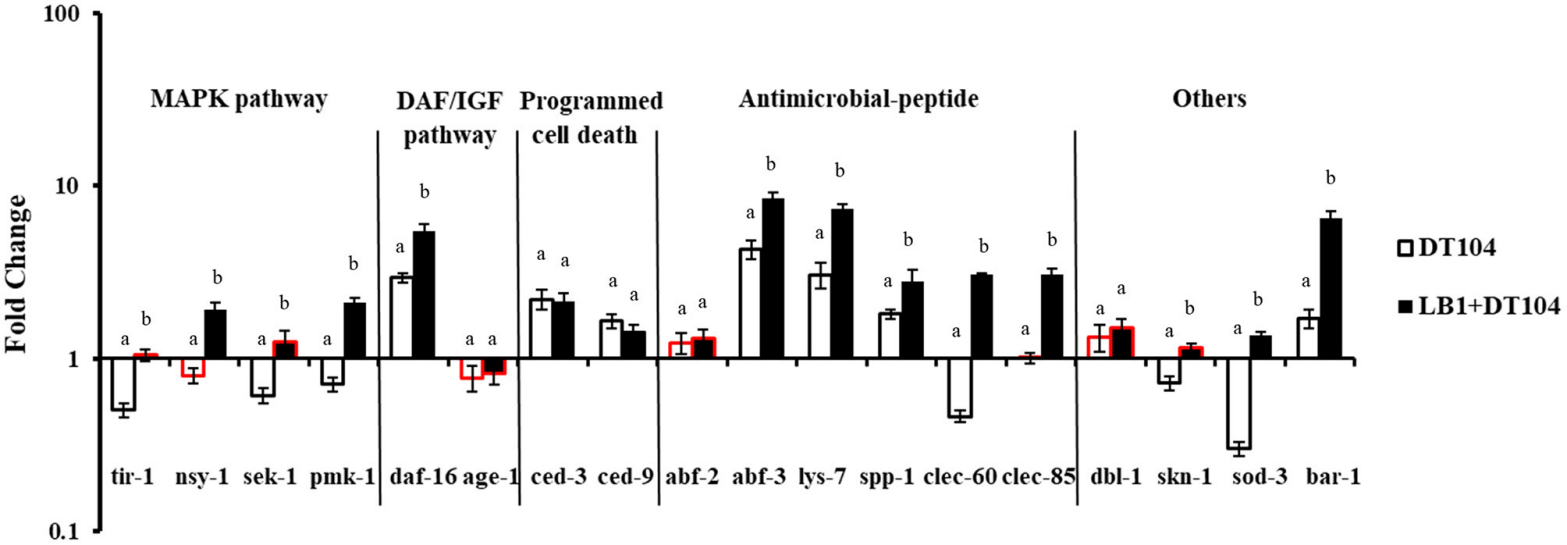
Expression of selected genes in *C. elegans* relevant to the host response to *Salmonella* infection and the protection from *L. zeae* LB1. The worms were sampled on day 2 of the life-span assay. The baseline is the level of gene expression of *C. elegans* on OP50. Relative expression was determined using the 2^−ΔΔ^Ct method as the ratio of transcription level of the treatment group to control group and expressed as fold changes. Data are presented as mean ± S.D. The bar border labeled with red color had no significant difference (*P* > 0.05) for the same gene between the treatment group (DT104 infected) and the control group (OP50 fed), while the remaining without red color differed significantly between the DT104 infected group and the OP50 fed group (*P* ≤ 0.05). Means marked with “a”, “b” were significantly different (*P* ≤ 0.05) for the same gene between the treatment group (LB1 + DT104) and the group infected with DT104 only.

As shown in Figure 2, while the gene expression of *nsy-1, age-1, abf-2, clec-*85, and *dbl-1* in the nematode infected with DT104 showed no significant changes (*P* > 0.05), the expression of *daf-16, ced-3, ced-9, abf-3, lys-7, spp-1*, and *bar-1* genes was upregulated (*P* ≤ 0.05) compared with uninfected worms. In contrast, the expression of most selected genes associated with the p38 MAPK pathway (*tir-1, sek-1*, and *pmk-1*), *clec-60* for antimicrobial peptide production, and *skn-1* and *sod-3* for other defense molecules was decreased (*P* ≤ 0.05). Compared with the nematode infected with DT104 only, pretreatment of the worms with *L. zeae* LB1 significantly upregulated (*P* ≤ 0.05) the expression of almost all the selected genes except for *age-1, ced-3, ced-9, abf-2*, and *dbl-1*.

### Involvement of Cell Signaling and Production of Antimicrobial Peptides and Other Defense Molecules in the *C. elegans* Resistance to DT104 Infection

To determine how cell signaling and production of antimicrobial peptides and other defense molecules affect the resistance of *C. elegans* to DT104 infection, the life-span of 14 different mutants infected with DT104 was investigated in comparison with the WT. The mutants included ag3 (defective in *nsy-1*), ag1 (*sek-1*), and km25 (*pmk-1*) defective in the p38 MAPK pathway; mu86 (*daf-16*) and hx546 *(age-1*) defective in the DAF/IGF pathway; mutants n717 (*ced-3*), n1950 (*ced-9*), and the double mutant n2812/n717 (*ced-9*;*ced-3*) defective in the PCD pathway; ok3366 (*abf-3*), ok1384 (*lys-7*), and ok2703 (*spp-1*) defective in antimicrobial peptide production; and nk3 (*dbl-1*), gk235 (*sod-3*), and zu67 (*skn-1*) defective in the production of other molecules with a defense function.

**Figure 3:**
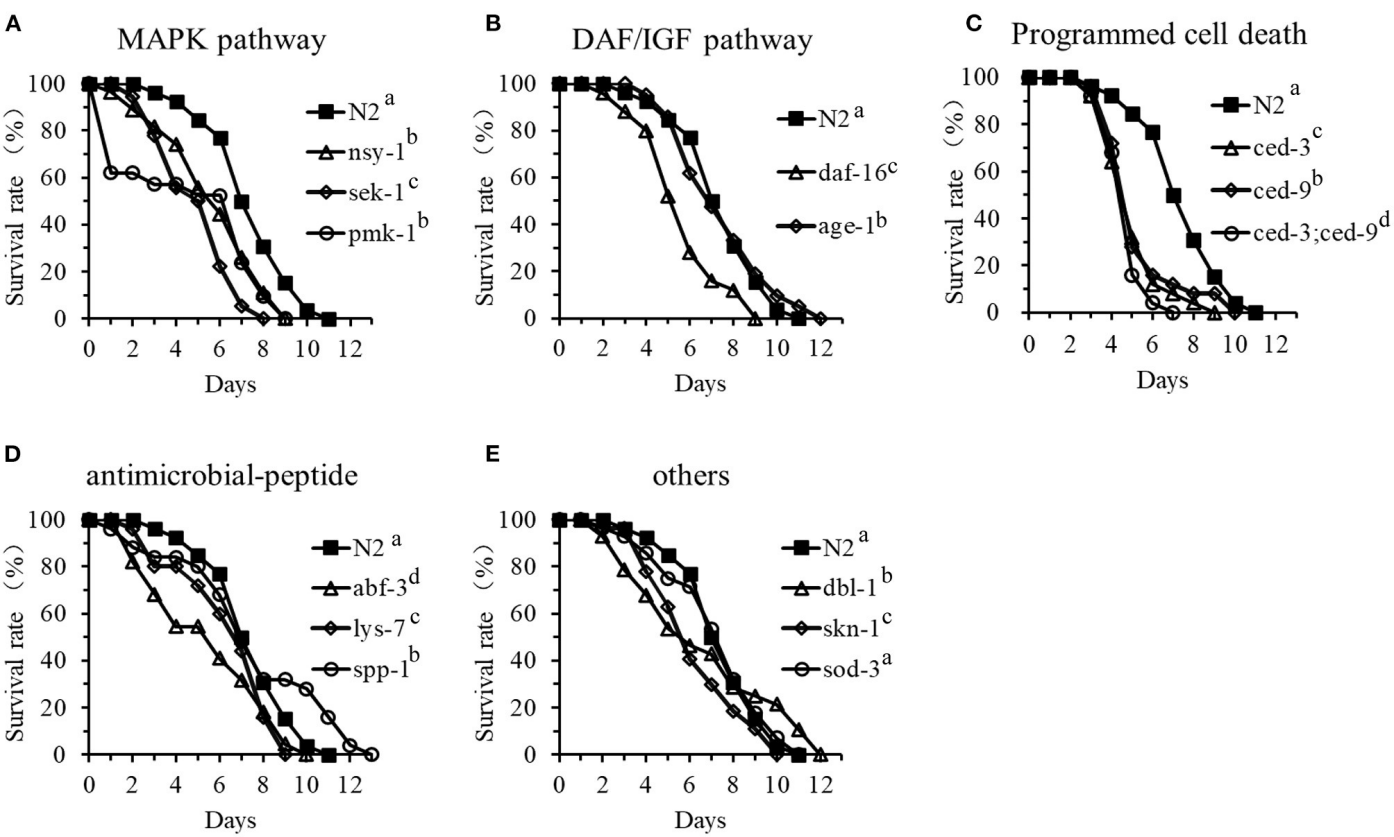
The life-span assay of *C. elegans* mutants in comparison with the WT that are infected with *S*. *typhimurium* DT104. **(A)** Survival curves of p38-MAPK pathway mutants: *nsy-1(ag3), sek-1(ag1), pmk-1(km25)*; **(B)** Survival curves of DAF/IGF pathway mutants: *daf-16(mu86), age-1(hx546)*; **(C)** Survival curves of PCD pathway mutants: *ced-9(n1950), ced-3(n717)*, or *ced-9;ced-3(n1950;n717)*; **(D)** Survival curves of the mutants defective in antimicrobial peptide genes: *abf-3(ok3366), lys-7(ok1384)*, or *spp-1(ok2703)*; **(E)** Survival curves of other mutants with a defense function: *dbl-1(nk3), skn-1(zu67), sod-3(gk235)*. All treatments were feed with *salmonella* DT104 as food instead of *E. coli* OP50. All the groups showing different letters were significant different (*P* ≤ 0.05) in their survival curves.

The results showed that the life-span of the tested mutants ag3, ag1, and km25 defective in the p38 MAPK pathway was all significantly shorter than that of the WT nematode when exposed to DT104 (Figure 3A). Mutants n717, n1950, and n2812/n717 defective in the PCD pathway became more susceptible to DT104 infection (Figure 3C). The life-span of these mutants with one gene mutation was reduced by more than 30% compared to the WT. Furthermore, the mutant (n2812/n717) with two mutations (defective in both *ced-3* and *ced-9*) had only a half of life-span of the WT. For the remaining mutants, mutants mu86 (defective in *daf-16*), ok3366 (defective in *abf-3*), ok1384 (defective in *lys-7*), and zu67 (defective in *skn-1*) showed a shorter life-span (*P* ≤ 0.05) than the WT (Figures 3B,D,E). In contrast, the life-span of the mutants that are defective in *age-1* (hx546), *spp-1* (ok2703), or *dbl-1* (nk3) were more resistant to DT104 infection with an increased life-span compare with the WT nematode (Figures 3B,D,E). Mutant gk235 (defective in *sod-3*) was an exception, which had a similar life-span of the WT when exposed to DT104 (Figure 3E).

### Regulation of Cell Signaling and Production of Antimicrobial Peptides in *C. elegans* by LB1

**Figure 4:**
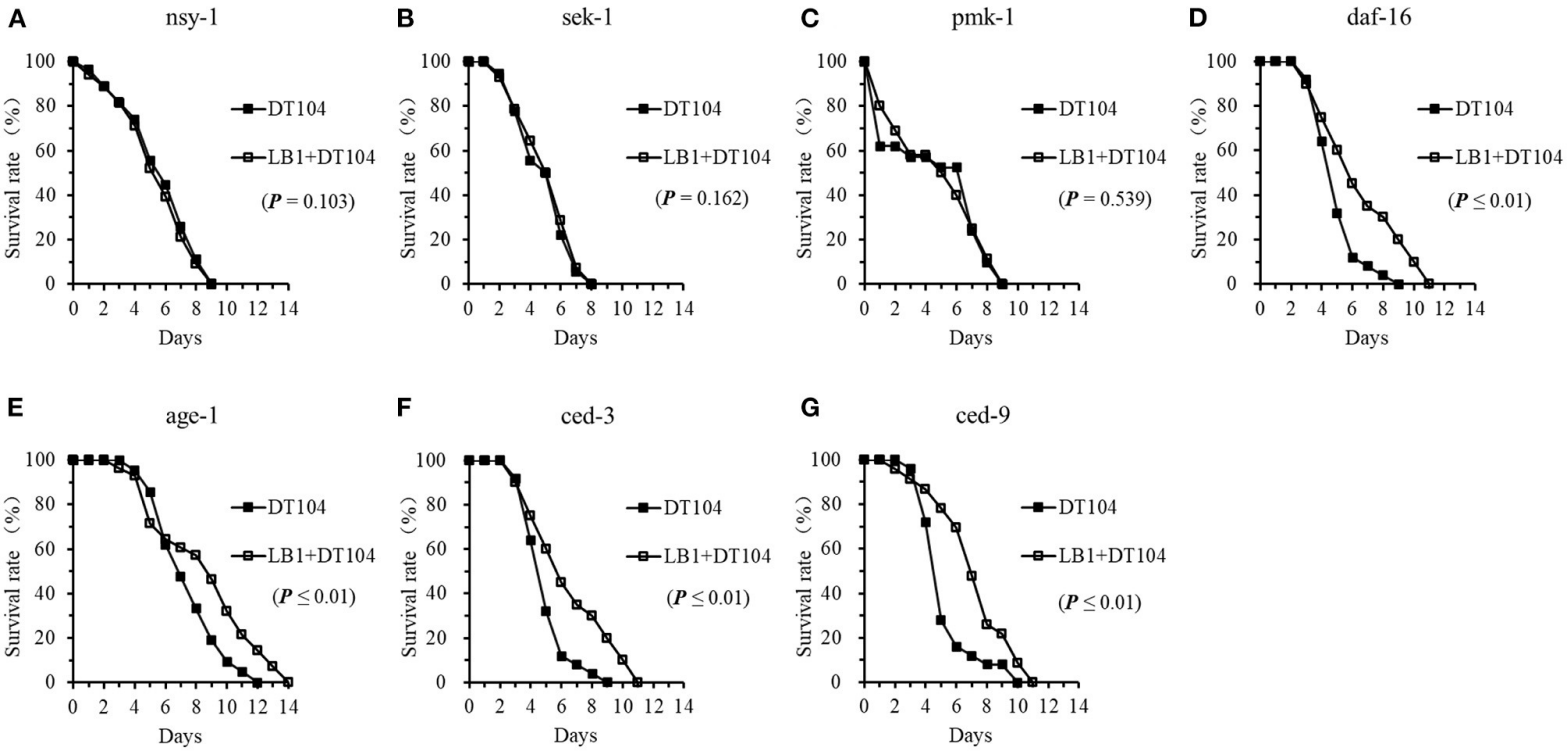
Effects of *L. zeae* LB1 on the resistance to *S*. *typhimurium* DT104 infection of *C. elegans* mutants that are defective in the genes for cell signaling. The life-span of different types of *C. elegans* mutants, including *nsy-1* **(A)**, *sek-1* **(B)**, *pmk-1* **(C)**, *daf-16* **(D)**, *age-1* **(E)**, *ced-3* **(F)**, and *ced-9* **(G)**, responding to DT104 infection were evaluated with or without the pretreatment of *L. zeae* LB1. All the assays were treated with 10^8^ CFU/ml LB1 or *E. coli* OP50 (control group) for 18h and then with DT104 for remain days. Comparisons were made between the worms pre-exposed to *L. zeae* LB1 prior to DT104 infection (LB1 + DT104) and those exposed to DT104 only (DT104). The *P* value for each comparison is indicated inside of each panel.

To determine the role of *L. zeae* LB1 in regulating the cell signaling of *C. elegans* to resist *S*. *typhimurium* DT104 infection, 7 mutants were examined for their resistance to DT104 infection after pretreatment with LB1. Interestingly, only the three mutants that are defective in *nsy-1, sek-1*, or *pmk-1* demonstrated no changes to DT104 infection even though they were pretreated with LB1 (Figures 4A–C), suggesting no protection from the isolate. These three mutants were shown to be more susceptible to DT104 infection than the WT in the life-span assay without LB1 pretreatment (Figure 3A). The mutants that are defective in *daf-16, ced-3*, or *ced-9* and were also shown to be more susceptible to DT104 infection than the WT in the previous life-span assay without LB1 pretreatment (Figures 3B,C), however, the mutants exhibited a significantly increased life-span (*P* ≤ 0.05) after the pretreatment with LB1 (Figures 4D,F,G). The pretreatment with LB1 also significantly increased (*P* ≤ 0.05) the life-span of the mutant defective in *age-1* (Figure 4E) that was more resistant to DT104 infection compared to the WT in the previous assay without LB1 pretreatment (Figure 3B).

**Figure 5:**
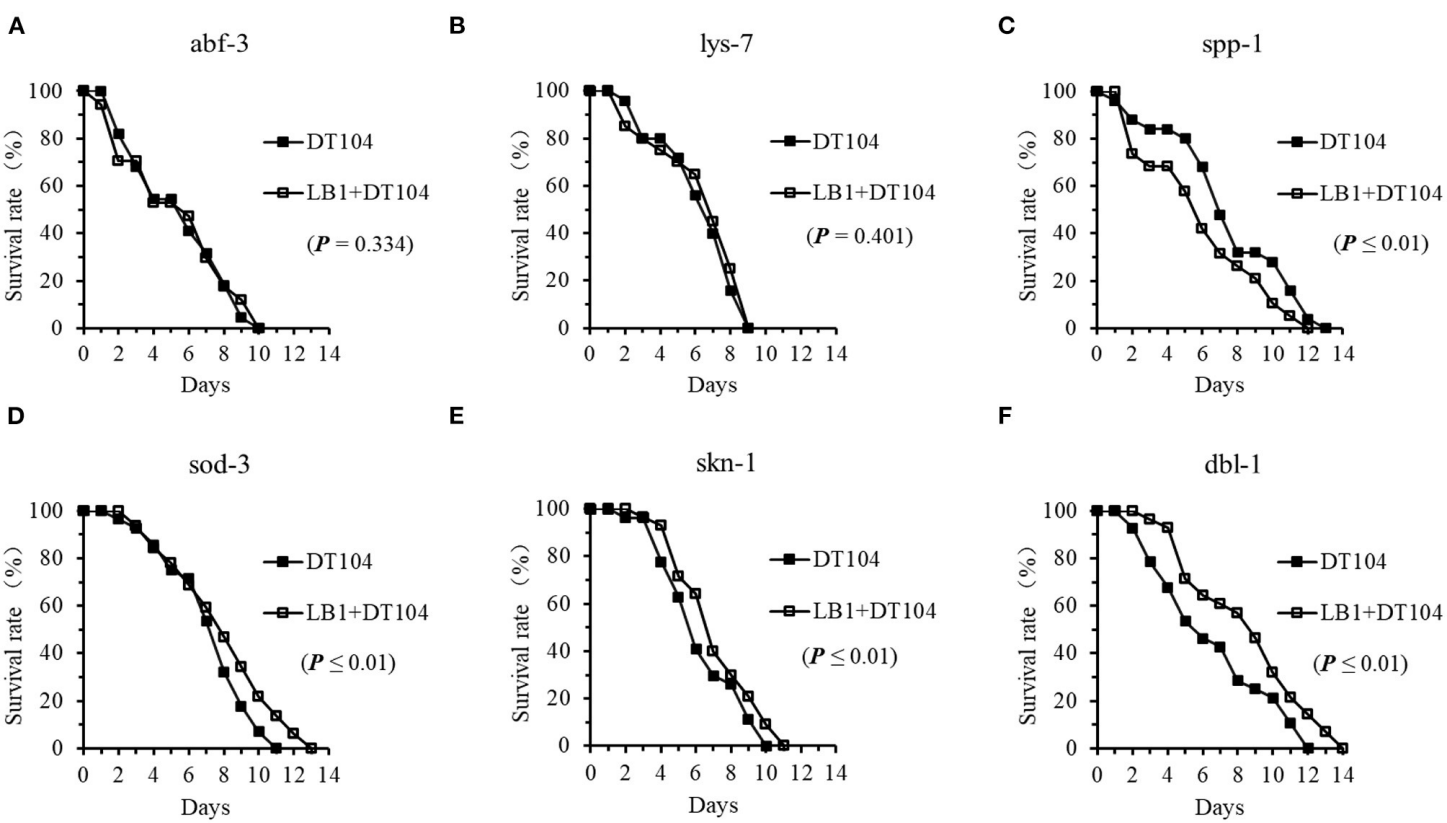
Effect of *L. zeae* LB1 on the resistance to *S*. typhimurium DT104 infection of *C. elegans* mutants that are defective in the genes encoding antimicrobial peptides or other molecules with a defense function. The life-span of different types of *C. elegans* mutants, including *abf-3(ok3366)* **(A)**, *lys-7(ok1384)* **(B)**, *spp-1(ok2703)* **(C)**, *dbl-1(nk3)* **(D)**, *skn-1(zu67)* **(E)**, *sod-3(gk235)* **(F)**, responding to DT104 infection were evaluated with or without the pretreatment of *L. zeae* LB1. All the assays were treated with 10^8^ CFU/ml LB1 or *E. coli* OP50 (control group) for 18h and then with DT104 for remain days. Comparisons were made between the worms pre-exposed to *L. zeae* LB1 prior to DT104 infection (LB1 + DT104) and those exposed to DT104 only (DT104). The *P* value for each comparison is indicated inside of each panel.

To determine if *L. zeae* LB1 regulates the production of antimicrobial peptides and other defense molecules in *C. elegans* responding to DT104 infection, the life-span of mutants ok3366 (defective in *abf-3*), ok1384 (defective in *lys-7*), ok2703 (defective in *spp-1*), zu67 (defective in *skn-1*), gk235 (defective in *sod-3*), and nk3 (defective in *dbl-1*) were investigated with or without LB1 pretreatment. As shown in Figure 5, both mutants defective in *abf-3* or *lys-7* had no changes in the life-span regardless of LB1 pretreatment, suggesting no protection from LB1 (Figures 5A,B). However, pretreatment with LB1 significantly increased (*P* ≤ 0.05) the life-span of the mutants that are defective in *sod-3, skn-1*, or *dbl-1* (Figures 5D–F) and showed no change, decreased or increased resistance to DT104 infection in the previous assay without LB1 pretreatment, respectively (Figure 3E). In contrast to these observations, the mutant defective in *spp-1* became more susceptible to DT104 infection after LB1 pretreatment (Figure 5C), which was more resistant to the infection without LB1 pretreatment (Figure 3D). In all the life-span assays with the mutants, the WT behaved similarly as in the previous assays with or without LB1 pretreatment.

## Discussion

The suitability of *C. elegans* for the studies of host immune response to bacteria including both foodborne pathogens and probiotics was further demonstrated recently (14, 34, 36–38). The nematode innate immune system is highly conserved and can be quickly activated through several immune regulatory pathways to protect the host against bacterial infection (7). Among them, the p38 MAPK and DAF/IGF signaling pathways play a vital role in combating bacterial infection in the intestine. For example, these two pathways were shown to be important in controlling *Salmonella* infection (10, 20). In addition, the PCD pathway was also found to have a role in the host resistance to *Salmonella* infection (23). Interestingly, we recently identified the positive role of both serotonin and dopamine in the host defense of *C. elegans* to *S*. *typhimurium* infection through regulation of the p38 MAPK and DAF/IGF pathways (21). To further elucidate this regulation, the present study has investigated the role of particular members in those pathways and provided new insight into the regulation of both cell signaling and production of antimicrobial peptides and other defense molecules in *C. elegans*.

In the present study, four genes (*tir-1, nsy-1, sek-1*, and *pmk-1*) in the p38 MAPK pathway, two genes (*daf-16* and *age-1*) in the DAF/IGF pathway, two genes (*ced-3, ced-9*) in the PCD pathway, six genes (*abf-2, abf-3, lys-7, spp-1, clec-60*, and *clec-85*) encoding antimicrobial peptides, and four other genes (*dbl-1, skn-1, sod-3*, and *bar-1*) reported previously with a defense function (26, 39, 40) were initially investigated for the possible involvement in the immune response of the WT nematode by examining their expression. When infected with *S*. *typhimurium* DT104, the expression of *daf-16, ced-3, ced-9, abf-3, lys-7, spp-1*, and *bar-1* genes was upregulated (*P* ≤ 0.05), whereas others such as *clec-60, skn-1*, and *sod-3* were suppressed. In particular, the expression of all the selected genes associated with the p38 MAPK pathway except for *nsy-1* was downregulated (*P* ≤ 0.05). These results suggested that the p38 MAPK pathway was one of the major targets by DT104 while the PCD and DAF/IGF pathways were also affected. The results from the life-span assay with various mutants supported the notion. The mutants that are defective in *nsy-1, sek-1*, or *pmk-1* (the major components in the p38 MAPK pathway), in *ced-3* or *ced-9* (the major components of PCD pathway), or in *daf-16* (a major component of DAF/IGF pathway) all became more susceptible to DT104 infection with over 20–30% reduction in the life-span compared to the WT (Figures 3A–C). Furthermore, the life-span of the double mutant defective in both *ced-3* and *ced-9* had only half of the life-span of the WT. Based on the data described above, it appears that the response of *C. elegans* to DT104 infection is mediated mainly through the regulation of the p38 MAPK, DAF/IGF, and PCD pathways.

Although many important innate immune pathways and effectors have been identified in *C. elegans*, there are differences in the host responses to different bacterial pathogens especially in the regulation of antimicrobial peptide production (28, 29, 31, 41). Analyses of mutations in different genes associated with the responses have identified downstream proteins involved in pathogen defense, such as LYS (lysozyme) family, ABF (*Ascaris suum* antibacterial factor) family, SPP (Caenopores are the saposin-like proteins) family, and C-type lectins family (29, 30). However, the role of these antimicrobial peptides in response to bacterial infection is yet to be fully elucidated (7, 14, 42). In the present study, the transcription of genes that encode for antimicrobial peptides including *abf-3, lys-7*, and *spp-1* was all significantly upregulated in the WT nematode when it was exposed to DT104. This suggests a vital role for antimicrobial peptides in the defense of *C. elegans* against *Salmonella* infection. This notion is also supported by the fact that *C. elegans* with a mutation in an antimicrobial peptide gene (*lys-7*, or *abf-3*) showed significant shorter life-span than the WT when the mutants were subjected to DT104 infection (Figure 3D). In our previous report, the mutant defective in *spp-1* showed shorter life-span than the WT when the nematode was subjected to ETEC infection only, but no changes in the life-span regardless of the pretreatment with *L. zeae* LB1 (34). However, in the current study the same mutant had significantly longer life-span than the WT when exposed to *S*. *typhimurium* DT104 only (Figure 3D), but a shorter life-span than the WT after the pretreatment with *L. zeae* LB1 (Figure 5C). While the reason underlying these observations is unclear, it provides another piece of evidence that the role of antimicrobial peptides can vary in the host response to different pathogens.

There were several reports recently that *Lactobacillus* can confer health benefits to *C. elegans* including life-span extending, protection against pathogen infection, and prevention from abiotic stress (20, 43–45). Park et al. (38) reported that probiotic *L. fermentum* strain JDFM216 stimulated the longevity and immune response of *C*. *elegans* through a nuclear hormone receptor (NHR) family and PMK-1 signaling (38). *Pediococcus acidilactici* P25 strain affected expression of the genes related to innate immune response and upregulated the abundance of transcripts in multiple pathways of *C. elegans*, including peroxisome, longevity, and MAPK pathways (42). Nevertheless, these reports have not yet identified immunomodulatory effects of *Lactobacillus* on the targeting sites downstream of the p38 MAPK and DAF/IGF pathways. Our previous study found that a selected strain *L. zeae* LB1 could provide protection by regulating *C. elegans* cell signaling through the p38-MAPK and DAF/IGF pathways to control the production of antimicrobial peptides and defense molecules (34). The same strain was also investigated for its immune-regulatory effects against *S*. *typhimurium* DT104 infection in the present study. The data from both the gene expression and life-span studies indicate that the protection offered by *L. zeae* LB1 against *Salmonella*-caused death also involved the p38 MAPK, DAF/IGF, and PCD pathways as well as the production of antimicrobial peptides and other defense molecules in *C. elegans*. In particular, five genes including *nsy-1, sek-1, pmk-1, abf-3*, and *lys-7* in the p38 MAPK pathway or for antimicrobial peptide production appear to be the sites regulated by LB1 as LB1 pretreatment provided no protection to the corresponding mutants (Figures 4A–C, 5A,B). Kim et al. (20) reported that *L. acidophilus* NCFM activates the p38 MAPK pathway (via TIR-1 and PMK-1) and the β-catenin signaling pathway (via BAR-1) against Gram-positive bacteria while it has no effect on Gram-negative (*P. aeruginosa* or *S. typhimurium*) (20). Very recently, we demonstrated that the protection of *L. zeae* LB1 against *S*. *typhimurium* DT104 infection was mediated by dopamine through both the p38 MAPK and DAF/IGF pathways (21). These observations with the data described in the present study suggest that the protection offered by *Lactobacillus* can be species or even strain-specific with different immunomodulation mechanisms.

**Figure 6:**
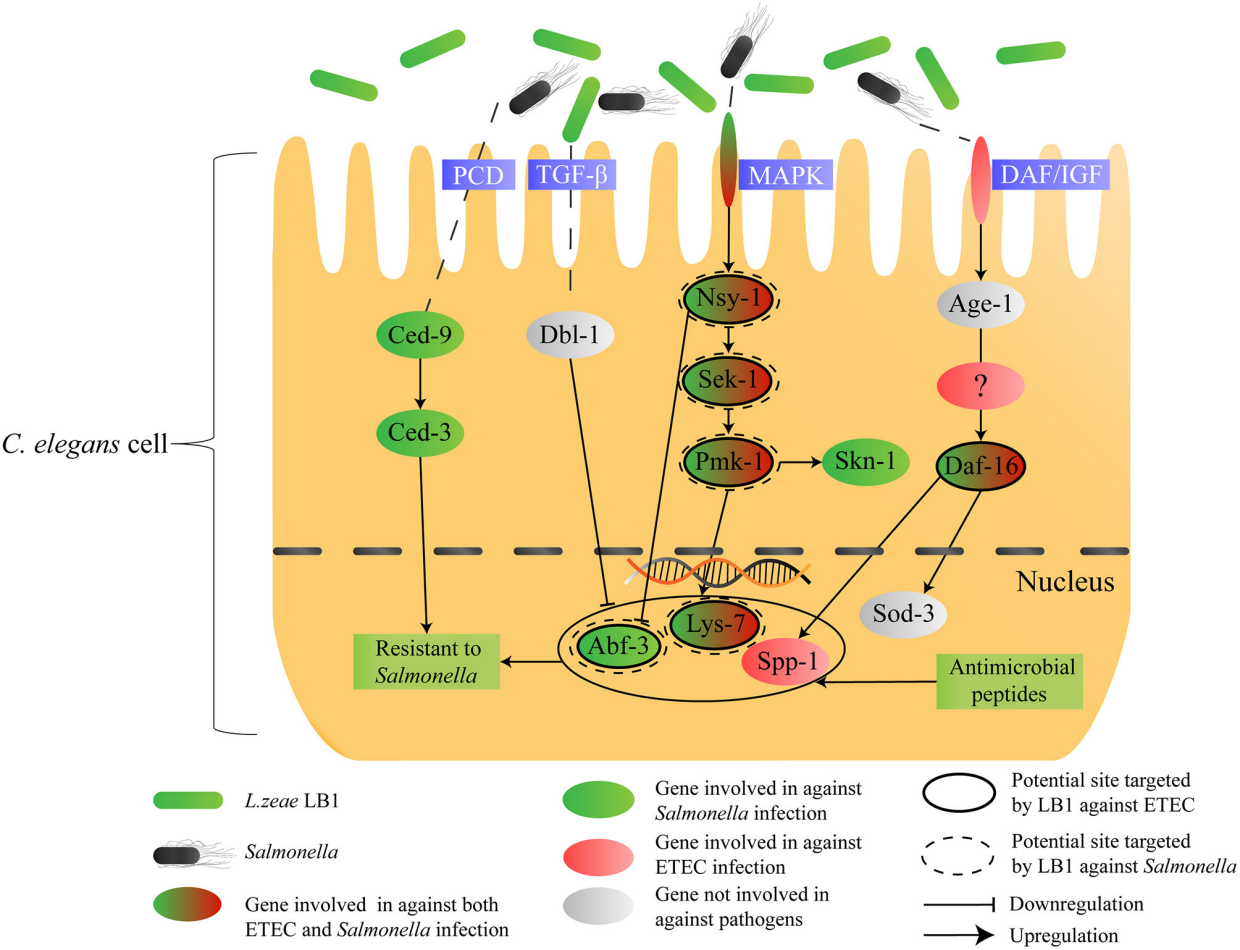
Schematic diagram speculating the immunomodulatory mechanism by *L. zeae*. The hypothesis was based on the data from both life-span assays and gene expression experiments of various *C. elegans* mutants either pretreated or not with *L. zeae* LB1 before *S*. *typhimurium* DT104 infection in the present study and on the data published previously on the regulation of *C. elegans* cell signaling by *L. zeae* LB1 against ETEC infection (34).

In conclusion, the present study has revealed: [1] the host response of *C. elegans* to *S*. *typhimurium* DT104 infection mainly involves the p38 MAPK, DAF/IGF, PCD pathways and production of antimicrobial peptides and defense molecules; [2] Nsy-1, Sek-1, and Pmk-1 (MAPK pathway) as well as Lys-7 and Abf-3 (antimicrobial peptides) appear to be the sites regulated by *L. zeae* LB1, leading to the protection; [3] *L. zeae* LB1 can induce different immune responses in *C. elegans* when the nematode is infected by different pathogens. To summarize the findings from the present study, a schematic diagram has been generated (Figure 6), which also proposes a possible immunomodulatory mechanism by *L. zeae*.

## Data Availability Statement

The original contributions presented in the study are included in the article/supplementary material, further inquiries can be directed to the corresponding author/s.

## Author Contributions

MZ and XL performed the experiments. MZ, HY, XL, and JG analyzed data. MZ and JG wrote the manuscript. MZ, HY, and JG designed the experiments. JG conceived the research. All authors approved the final version of manuscript.

## Conflict of Interest

The authors declare that the research was conducted in the absence of any commercial or financial relationships that could be construed as a potential conflict of interest.
